# Giant left ventricular pseudo-aneurysm

**DOI:** 10.1093/ehjcr/ytag393

**Published:** 2026-05-27

**Authors:** Rémi Deleuse, David Vancraeynest, Bernhard L Gerber

**Affiliations:** Department of Cardiovascular Diseases, Cliniques Universitaires St.Luc, and Pôle de Recherche Cardiovasculaire (CARD), Institut de Recherche Expérimentale et Clinique IREC UCLouvain, Av Hippocrate 10/2806, B-1200 Woluwe St. Lambert, Brussels, Belgium; Department of Cardiovascular Diseases, Cliniques Universitaires St.Luc, and Pôle de Recherche Cardiovasculaire (CARD), Institut de Recherche Expérimentale et Clinique IREC UCLouvain, Av Hippocrate 10/2806, B-1200 Woluwe St. Lambert, Brussels, Belgium; Department of Cardiovascular Diseases, Cliniques Universitaires St.Luc, and Pôle de Recherche Cardiovasculaire (CARD), Institut de Recherche Expérimentale et Clinique IREC UCLouvain, Av Hippocrate 10/2806, B-1200 Woluwe St. Lambert, Brussels, Belgium

**Keywords:** Left ventricular pseudo-aneuvrysm, Acute STEMI, CMR, Cardiac CT, Echocardiography

A 53-year-old male, active smoker, with hypercholesterolaemia, and a history of B-cell lymphoma treated with anthracycline-based chemotherapy and autologous haematopoietic stem cell transplantation, had presented one year prior at another institution with acute chest pain, elevated troponin, and inferior Q waves without ST-segment elevation. Computed tomography (CT) was performed to exclude pulmonary embolism, but in retrospect, may have already demonstrated a lateral wall aneurysm (Panel A, arrow). Colchicine therapy was initiated for suspected perimyocarditis. Initial echocardiography showed posterior akinesia and aneurysm with mild LV dysfunction, and coronary angiography revealed chronic total occlusion of the first obtuse marginal artery. After nicotinic acid enhanced ^18^F-FDG-PET confirmed myocardial viability of the unruptured aneurysm (Panels B–C), percutaneous angioplasty and stenting was performed two months after symptom onset. Nevertheless, the patient experienced progressive heart failure with enlargement of the pouch and was referred to our centre where echocardiography demonstrated severe LV dysfunction with a large pouch involving the lateral and posterior LV wall (Panels D–E, Supplementary material online, *Videos S1*–S2), partially filled with thrombus (Th). Cardiac MRI with cine sequences and late gadolinium enhancement (Panels F–G, Supplementary material online, *Video S3*) revealed a massive pseudoaneurysm (6.3 cm × 4.7 cm; volume 700 mL) secondary to contained rupture of the lateral free wall, with associated mural thrombus. Findings were corroborated by cardiac CT (Panels E and F). The patent underwent successful surgical repair with a prosthetic patch.

This case illustrates the insidious progression of a misdiagnosed posterolateral myocardial infarction into a contained LV free-wall rupture and pseudoaneurysm. Delayed revascularization and prior anthracycline exposure may have been possible contributing factors. It underscores the need for a high index of suspicion for MI in patients with chest pain and wall motion abnormalities, even when an inflammatory aetiology appears plausible, and highlights the essential role of early revascularization in preventing this life-threatening complication.

**Figure:**
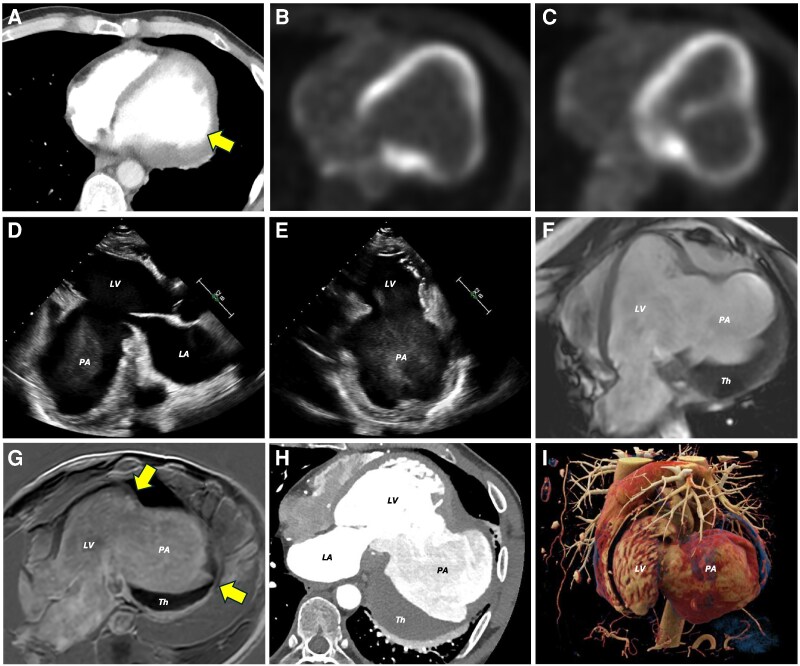


## Supplementary Material

ytag393_Supplementary_Data

## Data Availability

Data cannot be shared for ethical/privacy reasons.

